# Shortcomings of applying data science to improve professional football performance: Takeaways from a pilot intervention study

**DOI:** 10.3389/fspor.2022.1019990

**Published:** 2022-10-12

**Authors:** Mat Herold, Matthias Kempe, Ludwig Ruf, Luis Guevara, Tim Meyer

**Affiliations:** ^1^Institute of Sports and Preventive Medicine, Saarland University, Saarbrücken, Germany; ^2^Deutscher Fußball-Bund, Frankfurt am Main, Germany; ^3^Center for Human Movement Sciences, University Medical Center Groningen (UMCG), University of Groningen, Groningen, Netherlands; ^4^TSG ResearchLab gGmbH, Zuzenhausen, Germany; ^5^D.C. United, Washington, DC, United States

**Keywords:** soccer (football), performance analysis, data science, coaching (performance), passing ability, soccer analytics

## Abstract

Positional tracking data allows football practitioners to derive features that describe patterns of player behavior and quantify performance. Existing research using tracking data has mostly focused on what occurred on the pitch, such as the determinants of effective passing. There have yet to be studies attempting to use findings from data science to improve performance. Therefore, 24 professional players (mean age = 21.6 years, SD = 5.7) were divided into a control team and an intervention team which competed against each other in a pre-test match. Metrics were gathered via notational analysis (number of passes, penalty box entries, shots on goal), and positional tracking data including pass length, pass velocity, defensive disruption (D-Def), and the number of outplayed opponents (NOO). D-Def and NOO were used to extract video clips from the pre-test that were shown to the intervention team as a teaching tool for 2 weeks prior to the post-test match. The results in the post-test showed no significant improvements from the pre-test between the Intervention Team and the Control Team for D-Def (*F* = 1.100, *p* = 0.308, η^2^ = 0.058) or NOO (*F* = 0.347, *p* = 0.563, η^2^ = 0.019). However, the Intervention Team made greater numerical increases for number of passes, penalty box entries, and shots on goal in the post-test match. Despite a positive tendency from the intervention, results indicate the transfer of knowledge from data science to performance was lacking. Future studies should aim to include coaches' input and use the metrics to design training exercises that encourage the desired behavior.

## Introduction

To date, research on data science in football has primarily used observational designs to extract what occurred on the pitch, such as how opposing teams' centroid position is strongly related, especially in a forward and backwards direction (1), or that high risk passes occurring around the edge of the penalty box have the highest reward (2). Some experimental studies using data science have also been conducted that have produced findings applicable to the coaching process. For instance, discovering technical and physical differences between small-sided games (SSGs) and 11v11 match play (3), tactical differences between common playing formations (4) and some drawbacks associated with high-pressing (5). However, other than a few professional teams who publicly utilize positional data for in-house performance analysis, there is a paucity of research attempting to use positional tracking data to improve performance in competitive settings (6). The disconnect between research and practice, especially the lack of approaches using metrics and tools developed from data science was shown by a recent survey (7). In the survey, just 22 percent of 145 professional practitioners reported the use of such approaches in training with 35% of practitioners using KPIs (key performance indicators) for matches and only 19% using them for both training and matches. This leaves considerable room to investigate if and how data science can be applied in the training process to foster player education and development. Therefore, this pilot intervention study using professional football players is the very first attempt to close this gap.

Pilot studies are valuable in that they allow for reduced sample sizes and encourage participation in the applied setting, merging the path of researcher and practitioner (8, 9). Furthermore, pilot studies are more conducive to the rigorous, fast-paced environment within professional sports offering feasibility and a preview of methodological challenges for larger studies (10, 11). In this work, a lengthier intervention would have been desirable, especially considering that viewing tactical video (the nature of our intervention) has shown not to elicit mental fatigue nor impair subsequent physical and technical performance (12). In addition, more subjects and subsequent passes would have served to power the study and improve test-retest reliability. However, difficulty in recruitment and logistical constraints justify the sample size for this pilot study (13). Factoring in the normal team video sessions, players' pre-training routines, post-training individual meetings, weight training, etc., it was determined by the coaching staff that anything more would interfere with the team's objectives.

Earlier studies using positional tracking have mostly involved the examination of passing behavior. This focus is warranted (and feasible) as passing is the most frequent individual tactical action in a game (14) and therefore considered a key skill (15, 16). For instance, a greater number of passes, forward passes, and passes in the opposition's half of the field have shown to discriminate between winning, drawing and losing teams (17, 18). As such, we chose to focus on the improvement of passing effectiveness in this pilot intervention.

Studies using positional tracking data demonstrated effective passes force defenders out of position, creating space that leads to higher probability goal-scoring chances (19, 20). Perhaps this explains why winning teams have been shown to outplay more opponents with passes than losing teams (19, 21). In other words, passes that eliminate a greater number of defensive players increase the attacker's space control in front of the goal and can be ranked as effective (22). These increases in spatial dominance and outplaying defenders with passes had a positive effect on the number of goals scored and the chances of winning a game. Following the presented results, we chose to use the number of outplayed defenders (NOO) as one of the metrics to evaluate passing performance in this study.

Along with passes that outplay opponents in a vertical direction, sideways and backwards passes can force the defense to shift, leaving gaps between defenders. Considering the importance of unbalancing the defense, (23) calculated a defensive disruptiveness score (D-Def: an aggregated variable to quantify passing solely based on tracking data) as an index that represents the change in defensive organization resulting from a pass (24). The D-Def metric could distinguish top, average, and low performance passes by comparing D-Def, pass length, pass angle, and pass velocity in the top 10%, average 80% and bottom 10% passes ranked on D-Def score. Consistent with the findings of Chassy (25) the speed and precision of passes are predictors of success, corresponding to greater D-Def scores. Therefore, in addition to the number of outplayed opponents, D-Def was the second data driven metric used to measure the effectiveness of each pass.

One method utilized by most professional football teams to improve individual and team performance, including passing performance, is video analysis in the match planning and development of players (26, 27). Video analysis offers coaches the opportunity to use pre-selected clips to assess performance and gives players the chance for critical self-appraisal (28). Reflective practice using video feedback has been demonstrated to be a useful tool to improve several cognitive components such as game understanding and decision-making in football (29). In the training of goalkeepers, the addition of video feedback (observing one's own performance) had significant effect on improving performance compared to video modeling (observing an expert perform the skill) alone (30).

Despite being common in practice, research on video analysis in football has mostly covered practitioners' perceptions of video analysis (31) and how it is used in their daily work (26, 32). Therefore, further examination of the role of video feedback on performance outcomes would be a worthwhile purpose for research in football. Moreover, no studies have attempted to combine information gathered via positional tracking data and transform it into an educational tool to demonstrate effects beyond laboratory tasks, in real competitive situations. Therefore, this pilot study aims to examine the effectiveness of video feedback consisting of positive and negative examples of players' passes of two metrics—D-Def and number of outplayed opponents (NOO)—for the performance of individual and team passing performance. It is hypothesized that players on the intervention team will show significantly greater improvement in D-Def and NOO in the post-test match.

## Methods

### Participants

Twenty four professional football players participated in this study (mean age = 21.6 years, SD = 5.7).

All participants were rostered on a USL Championship team considered the United States 2nd Division in which they practice about 8 to 15 h a week. At the time of the intervention, the team had been playing together for a duration of 6 months. The present research fully complies with the highest standard of ethics and participant protection which followed the guidelines stated in the Declaration of Helsinki (2013) and was approved by the ethics committee of Saarland University (registration number 2573003). All participants gave their written informed consent; parental consent was provided for players younger than 18 years of age.

### Procedure

Pre and post-test matches consisting of 11v11 were played with each game lasting two 15 min halves with a 3 min half-time period. Based on position, players were randomly selected to either control team or the experimental team. Both teams were evenly matched by coaches and were instructed to play in a 4-2-3-1 formation.

**Figure 1:**
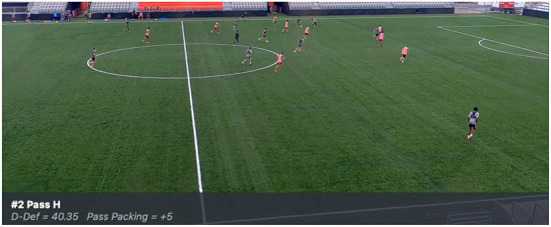
Example of how players received video feedback for each pass on D-Def and Outplayed Opponents (“Pass Packing”).

**Figure 2:**
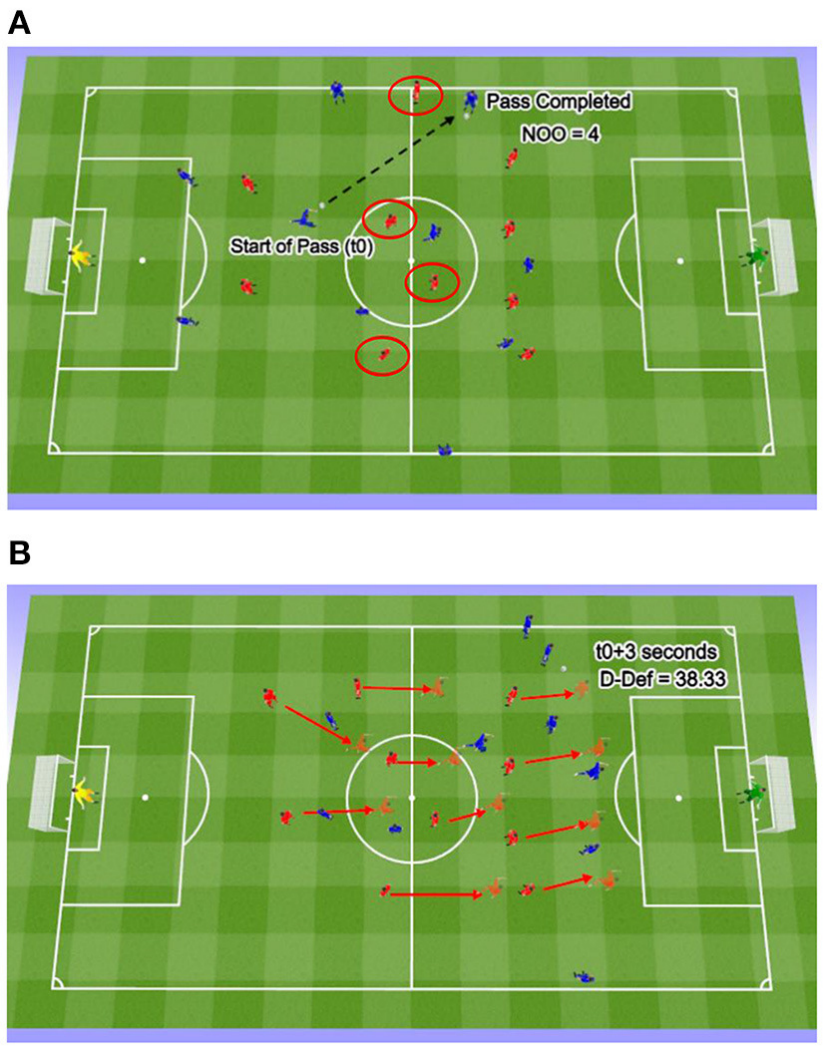
A 2-dimensional representation of how D-Def and NOO were determined by a pass. The blue team is attacking toward the right against the defensive team in red. In **(A)** from the start of the pass (t0) to the time of pass completion, 4 defensive players (circled in red) were eliminated in the longitudinal direction. In **(B)** the red arrows represent the displacement of the defense from t0 to t+3 s, yielding a D-Def score of 38.33.

Following the pre-test match, the experimental team was shown video clips of six examples of passes with a low D-Def score (<mean) and six examples of passes with a high D-Def score (>mean) prior to joining the team for training sessions. The control team did not receive any intervention. During the video intervention session, each of the 12 passes was shown three times and the D-Def score as well as the Number of Outplayed Opponents were visible for players prior to and during each pass (see Figure 1). Thus, the Intervention Team viewed 216 passes in throughout the duration of the intervention with each of the six intervention sessions lasting ~15 min in duration. During the video sessions and throughout the intervention, no coaching or feedback was given other than an initial explanation of how the D-Def metric and NOO metrics work and identifying each pass both orally and visually (Figure 2). Players were neither encouraged nor discouraged from discussing the video and/or their passes amongst themselves. The video played for 10 s prior to the execution of the pass and 10 s after the pass was completed to provide players with adequate game context. This meant players could identify positive and negative behavior based on these numbers and the effect of each pass on their own. Showing both positive and negative examples was the chosen method as individual players respond differently to various forms of feedback (29). At the conclusion of the intervention, the teams competed in an identical re-test and were evaluated again for their performance on the metrics. A placebo video was given consideration but since the professional players on the team involved have daily team video sessions lasting between 10–45 min it was deemed unnecessary.

### Data collection

The pre and post-test matches were monitored and recorded via the camera at Segra Field in Leesburg, VA, provided by Spiideo (https://www.spiideo.com). Segra Field was used as it serves as both the training and match field for the participating team. To evaluate the performance of a team according to the tactical principles and analyse the relationship between tactical performance and match outcome and to assess the success of the chosen intervention (see below), positional tracking data was collected and processed. Players were tracked with a semi-automatic optical tracking system (STAT Sports; STATS LLC, Chicago, IL) that captures the X and Y coordinates of all players at 10 Hz. Every pass was tagged manually in the Spiideo application to the nearest 0.1 of a second for the moment the ball was passed to the moment the ball was received. Both the tracking data and the ball event data were then imported as individual data frames in Python 3.6 and automatically processed on a match-by-match basis.

### Metrics

D-Def—computed as the displacement of the average X and Y positions (or centroids) for the full team, and the defensive, midfield, and attacking lines between the moment a pass was given (t0) and 3 s later (t0+3). D-Def is constructed by three components: the disruption in the longitudinal and lateral directions, and disruption of the team surface and spread area [For an in-depth description, see (33)]. This results in a measure from 0 to 150 (with 0 indicating no disruption and 150 indicating a maximum of disruption).

Position data was also used to calculate the number of outplayed opponents (NOO) by determining the difference in opposing players between the ball carrier and target goal from the moment each pass is played to when it was received (19, 20). Therefore, NOO could range between−10 (10 more player between the original position and the goal) to 10 (10 less players between the original position and the goal).

### Statistical analysis

Statistics for all passing-related performance metrics of the two teams were compared with one another for both the pre-test and the post-test matches. In the post-test match, four players (40%) from the control team and one player (10%) from the intervention team missed with one player out due to illness, two players got injured during the length of the intervention, and two players were called up to the 1st team. Therefore, after examining various approaches to handle missing data, we utilized a principled method by inputting a weighted nearest neighbors' approach in SPSS statistical software (34, 35). D-Def and NOO are only applicable for completed passes and therefore, the sample comprised of 187 completed passes (95 pre-test and 92 post-test) in the control team and 184 total passes (76 pre-test, 107 post-test) for the intervention team.

Pre and post-test match team comparisons were made for the main dependent variables including D-Def and Number of Outplayed Opponents and descriptive analysis was completed for Number of Passes, Penalty Box Entries, Shots on Goal, Pass Length, and Pass Velocity.

Data for D-Def and Number of Outplayed Opponents were normally distributed for each factor combination based on a Shapiro-Wilk test. A 2 × 2 repeated measures ANOVA (timepoint × team) was used to test for interactions, main effects, and simple main effects for timepoint (pre- vs. post-test matches) and team (Intervention Team vs. Control Team) for D-Def and NOO with an alpha level of.05. Effect sizes were calculated using partial eta squared (η^2^) with 0.14 or greater representing large effects, 0.06 or greater as medium effects, and 0.01 or more as small effects (36). All statistical tests were carried out with the statistical software IBM SPSS Statistics Version 25. An a priori power analysis was conducted using G^*^Power version 3.1.9.7 (37) to determine the minimum sample size required to test the study hypothesis. Results indicated the required sample size to achieve 80% power for detecting a medium effect, at a significance criterion of α = 0.05, was *N* = 96 for a 2 × 2 repeated measures ANOVA. Thus, the obtained sample size of *N* = 24 players was low in statistical power. However, given the number of observations including 371 completed passes and the contextual and environmental constraints associated with high-performance sport, the sample size for this pilot study can be based on feasibility (38).

## Results

**Figure 3:**
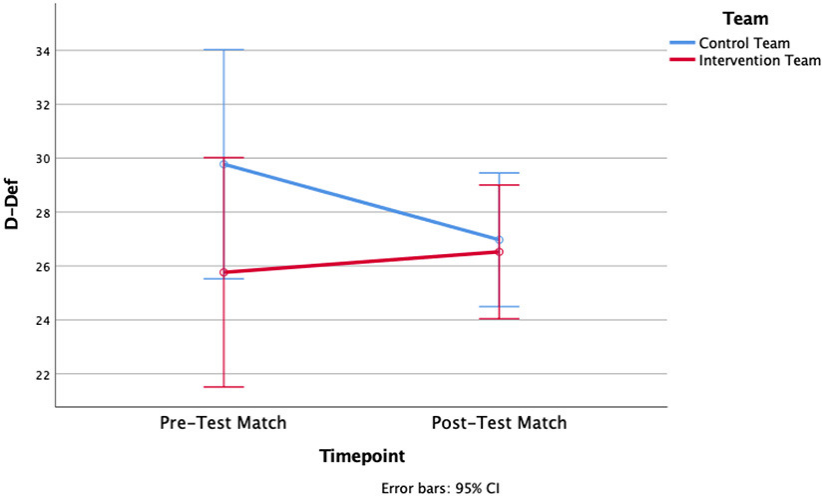
Differences in D-Def scores between Pre-and Post-test matches for the Control Team and the Intervention Team.

**Figure 4:**
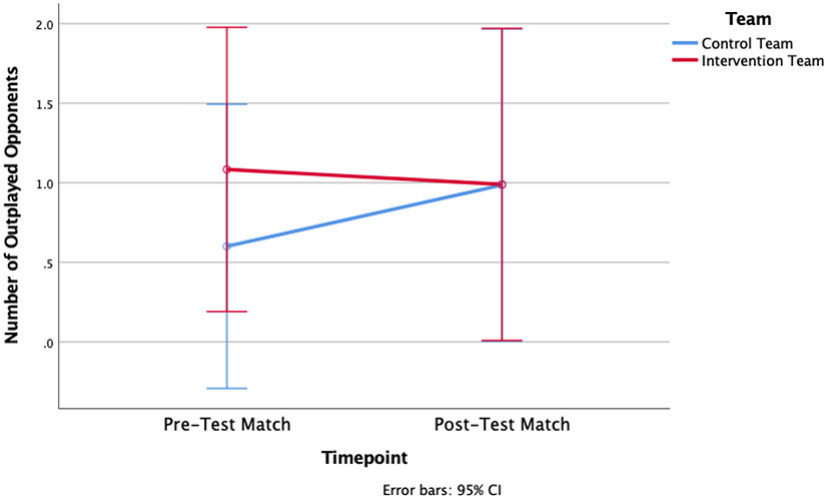
Differences in Number of Outplayed Opponents (NOO) between Pre-and Post-test matches for the Control Team and the Intervention Team.

A two-way ANOVA revealed that there was not a statistically significant interaction detected between the effects of timepoint (pre-test vs. post-test) and team (Intervention Team vs. Control Team) for D-Def [*F*_(1, 31.73)_ = 1.100, *p* = 0.308, η^2^ = 0.058] or NOO [*F*_(1, 18)_ = 0.347, *p* = 0.563, η^2^ = 0.019]. Similarly, there was neither an overall effect for the factor timepoint [*F*_(1, 10.47)_ = 0.363, *p* = 0.554, η^2^ = 0.02 for D-Def; *F*_(1, 18)_ = 0.128, *p* = 0.725, η^2^ = 0.007 for NOO] nor for the factor team [*F*_(1, 18)_ = 1.905, *p* = 0.184, η^2^ = 0.096 for D-Def; *F*_(1, 18)_ = 0.254, *p* = 0.620, η^2^ = 0.014 for NOO] (Figures 3, 4). As seen in Table 1, mean differences indicate that the Control Team's D-Def score decreased by 2.8, and their NOO score increased by 0.39. For the Intervention Team, their D-Def score increased by 0.76, but their NOO score decreased by an average of 0.09.

**Table 1:** Team averages between pre-test and post-test matches.

	**Pre-test control**	**Post-test control**	**Pre-test intervention**	**Post-test intervention**
D-Def	29.78 ± 6.46 95% CI = [25.52, 34.03]	26.97 ± 4.28 95% CI = [24.49, 29.45]	25.76 ± 6.34 95% CI = [21.51, 30.02]	26.52 ± 3.1 95% CI = [24.04, 29.00]
Number of outplayed opponents	0.6 ± 1.68 95% CI = [−0.29, 1.49]	0.99 ± 1.57 95% CI = [0.01, 1.97]	1.08 ± 0.89 95% CI = [0.19, 1.97]	0.99 ± 1.38 95% CI = [0.01, 1.97]
Number of passes	9.3 ± 6.63	7.4 ± 4.25	7.4 ± 4.38	10.3 ± 4.42
Penalty box entries	8	8	2	11
Shots on goal	3	2	2	4
Average pass length (meters)	19.53 ± 10.0	20.57 ± 12.23	16.38 ± 6.62	19.12 ± 11.60
Average pass velocity (m/s)	12.4 ± 4.53	11.98 ± 4.13	10.97 ± 3.47	10.96 ± 4.35

As seen in Table 1, the Intervention Team showed greater numerical increases for Number of Passes, Penalty Box Entries, Shots on Goal, and Pass Length. In contrast, the Control Team only made a slight increase in Pass Length.

## Discussion

Disrupting the opponent's organization and outplaying opponents are important outcomes of effective passing. In theory, improving players' ability in these areas would increase their team's chances of scoring goals. Based on these assumptions, this study examined the use of a positional tracking, data driven video intervention in an experimental setting (11 vs. 11 football game) to investigate if well-established metrics of observational studies can be used to improve passing effectiveness.

In the present study, there were no significant differences found between the Control Team and the Intervention Team for either of the metrics gathered via positional tracking data: D-Def or Number of Outplayed Opponents. The improvements were only small, insignificant changes for D-Def (+0.76) and a slight decrease in NOO (−0.09) for the Intervention team. Given that the control team's D-Def score decreased without any presumed external influence, the difference can be interpreted as a lower boundary for the reliability of the measurement procedure.

Despite no significant improvements for D-Def or NOO, the Intervention Team made greater numerical increases than the Control Team in more traditional key performance indicators (7), including Number of Passes, Penalty Box Entries, Pass Length, and Shots on Goal. Broadly speaking, the greater numerical increases shown by the Intervention Team supports previous studies that a video intervention can lead to general improvements in performance. These findings are supported by research in other sports showing improved decision making (tennis) and tactical knowledge (volleyball) with the use of video feedback (39, 40).

The Intervention Team's execution of more passes by players more frequently positioned in attacking areas of the field and gain seven extra Penalty Box Entries in the post-test match compared to the pre-test match is in accordance with previous studies finding passes from the midfield into the final third lead to greater penalty box possessions (41, 42). This could be the result of the video intervention as D-Def and NOO show the highest values for these types of passes. Thus, the players of the intervention team might have prioritized these passes based on the information presented in the video clips. These results suggest in future studies it could be of interest to not only measure single passes, but sequences of passes to capture the combined effects of passes of different lengths, velocities, and vectors.

The failure to achieve significant improvement for D-Def and NOO could be due, in part, to low sample size, as well as limitations of transferability from the chosen form of video feedback to on-field performance. One aspect that may have limited the transfer to the field was the speed in which the video was played back. For example, video feedback on kicking performance and temporal patterns in U-10 players discovered only the slow-motion video group elicited significant improvements whilst the video played at normal speed did not (43). Though in this study professional players were used, and the objective was to improve tactical performance more so than technical performance, use of slow-motion replay could have elicited greater improvements. Each pass was shown three times, but at normal playback speed the Intervention Team may have been unable to pick up on enough of the match context to determine what led to a specific pass having a higher or lower score.

Along the lines of decision making in football, one could argue that D-Def is too multifaceted and/or complex for players to consider in the chaos in a game. Although a player may comprehend that longer passes traveling at a greater velocity, in a slightly more forward direction cause the greatest disruption to the defense (24), players must make rapid decisions relative to the match context. For instance, passing decisions are largely influenced by teammates' movement and positioning relative to the ball carrier as well as the organization of the defense; two factors which determine open passing lanes (44). On the other hand, players in the Intervention group performed worse in the post-test match for NOO, a rather simple concept of subtraction. Further, well-known football terminology (and a popular metric called *Packing rate*) such as *breaking defensive lines*, and *penetrating passes*, exist, to illustrate the passing-related principle of play. Therefore, other limitations of the intervention must be considered.

One factor that could have limited transfer was the lacking coaches' involvement in the video education process. Thus, the onus was on the players' ability to reflect and their self-awareness, two prerequisites for learning (45), to understand when they made good passes and/or how they could have made better passes. In this study, the feedback about each pass was quantitative as players were simply shown a number indicating the D-Def and NOO value of each pass. It is possible that a more qualitative assessment, such as a “debate-of-ideas” would have been more likely to pay dividends in performance on the field (46). Albeit the effectiveness of discussion in the video feedback process depends on a high level of trust between athletes and coaches (47), the absence of open forum dialogue in this study could have limited decision making progress.

Besides more qualitative feedback from coaches, this study did not involve any exercises or drills on the field that could have enhanced player understanding and execution of the principles behind D-Def and NOO. Other intervention studies have involved specific training approaches with positive results. For example, a non-linear training approach (manipulating interacting constraints between the learner, task and environment) was found to improve decision making and actions (48) and the integration of differential learning was found to enhance creative and tactical behavior (49). Ultimately, information derived by data science would not be limited to video analysis and ideally, it would stimulate discussions between match/performance analysts and coaches to find ways to improve training and match tactics. It is recommended that prospective research combines findings from data science into training exercises that underscore the perceptual-action relationships of the chosen metric/s. These studies could follow the lead of previous studies where researchers collaborated for multiple weeks with coaches to create the implemented training program (48, 50).

Finally, the length of the intervention process was also short in comparison to other studies. With the team involved being in the middle of their professional season, the intervention was only able to be applied for a total of six sessions due to logistics and the preference of the coaching staff. Previous studies showed the importance of including more than twelve sessions (50, 51) and increased results with more sessions (52). Thus, future work on the integration of data science to performance is advised to give attention to the length of education process.

In conclusion, this pilot intervention study made the first attempt to gain a better understanding about integrating spatiotemporal data to improve football performance. Positive and negative examples of passes based on quantitative measures led to marginal improvements in D-Def but a slight decrease in NOO. While there was no significant improvement in the passing metrics, the intervention team's performance improved based on more traditional key performance indicators. Thus, there was an indirect effect of the intervention, and it can be assumed that football players may benefit from video feedback when attempting to improve passing performance. We think, this first pilot study shows that metrics derived from data science could improve player performance and improve tactical training, if, like in this study, metrics are well-explained to the players and data is processed quickly to facilitate the training. To continue bridging the gap between data science research and football practice, it is recommended future studies consider the length of the intervention, provide qualitative feedback, and include collaborative efforts between coaches and researchers to develop training sessions that reinforce any desired tactical behavior/s.

## Data availability statement

The raw data supporting the conclusions of this article will be made available by the authors, without undue reservation.

## Ethics statement

The studies involving human participants were reviewed and approved by the Ethics Committee of Saarland University (registration number 2573003). Written informed consent to participate in this study was provided by the participants' legal guardian/next of kin.

## Author contributions

MH and TM conceived and designed the analysis of this paper. MH and LG organized the study and collected positional data including the use of STATS Sports tracking devices. MK contributed to performing the analysis and integrating algorithms for D-Def and NOO. MH is the primary author with editing contributions from LR, MK, and TM. All authors contributed to the article and approved the submitted version.

## Funding

MH was supported by a Science and Health in Football scholarship funded by the Deutscher Fußball-Bund (DFB) and Saarland University.

## Conflict of interest

Author LR was employed by TSG ResearchLab gGmbH, Zuzenhausen, Germany. Author LR was employed by D.C. United. The remaining authors declare that the research was conducted in the absence of any commercial or financial relationships that could be construed as a potential conflict of interest.

## Publisher's note

All claims expressed in this article are solely those of the authors and do not necessarily represent those of their affiliated organizations, or those of the publisher, the editors and the reviewers. Any product that may be evaluated in this article, or claim that may be made by its manufacturer, is not guaranteed or endorsed by the publisher.
